# Reply to the comments on the letter of Peets et al. titled “Comment on solitons in the Heimburg–Jackson model of sound propagation in lipid bilayers are enabled by dispersion of a stiff membrane” by Drab et al

**DOI:** 10.1140/epje/s10189-023-00298-3

**Published:** 2023-05-30

**Authors:** Mitja Drab, Matej Daniel, Veronika Kralj-Iglič, Aleš Iglič

**Affiliations:** 1 Laboratory of Physics, Faculty of Electrical Engineering, University of Ljubljana, Tržaška 25, 1000 Ljubljana, Slovenia; 2 Department of Mechanics, Biomechanics and Mechatronics, Faculty of Mechanical Engineering, Czech Technical University in Prague, Technicka 4, 16607 Prague, Czech Republic; 3 Faculty of Health Sciences, Laboratory of Clinical Biophysics, University of Ljubljana, Zdravstvena Pot 5, 1000 Ljubljana, Slovenia; 4 Laboratory of Clinical Biophysics, Faculty of Medicine, University of Ljubljana, Vrazov Trg 2, 1000 Ljubljana, Slovenia

The goal of the original manuscript by Drab et al. [1] was to derive the otherwise contingent dispersion term in the Heimburg–Jackson (HJ) model [2] from first principles. The goal of the work was to present a model of axonal propagation that is comparable to the simplicity of the HJ model, but provides a physical background for the dispersion term presented in [2]. Dispersion terms that provide solitary wave solutions can be varied, and it is not immediately apparent that the fourth-order dispersion term used in [2] is the best choice or what physical principle underlies it hinges. The authors of [1] show that the fourth-order dispersion term follows naturally from the bending properties of the axon membrane.

In their comment Peets et al. discuss mainly methodological aspects of the study [1]. While some of the comments on the methods used in [1] (e.g., for the formal solution of differential equations) are relevant and useful, other more formalistic comments mostly do not contribute significantly to clarify better the basic physical assumptions of the model presented in [1] and previous models [2].

In the first part of the commentary, the authors discuss the differences between peakons and solitons. Drab et al. sought solutions for travelling waves of the form V = V(ξ), ξ = X − cT, where V is a function and c is the velocity of the moving frame, using the technique described, for example, in [3]. Drab et al. [1], on the other hand, were not aware of homoclinic orbits and their relation to solitary waves. Following the techniques presented in [2], the characterization of homoclinic orbits was not present in the calculation of solitary waves in [1]. The authors of [1] should therefore be grateful to Peets et al. for this insight.

J. P. Boyd defined three conditions for a solitary wave to be a soliton [4]. He considered a solitary wave to be a ‘soliton’ as a coherent structure that does not evolve in time due to a perfect balance between the steepening effects of nonlinearity and the propagating effects of wave dispersion. The authors of [1] were not fully aware of the distinction between ‘soliton’ and 'solitary wave' and used these two terms interchangeably. Since only the first two of the three conditions for a soliton were met in [1], it therefore seems reasonable to refer to solitons as solitary waves in [1]. It was not tested in [1] whether solitary waves can maintain their velocity and structure after interaction with another soliton, since this was not within the scope of the study in [1]. In any case, the authors of [1] should be grateful to Peets et al. for distinguishing the two terms (‘soliton’ and 'solitary wave').

The reference to the nonlinear coefficient seems to have nothing to do with the model described in [1], because as many experiments have shown, biological membranes can also exhibit nonlinear properties due to their internal macromolecular structure. Consequently, models that attribute a linear elastic structure to the membrane are only first approximations [5, 6]. The bending elasticity of the membrane is used to explain the dispersion in [1]. The Monge parameterization [7] of the bending elasticity of a membrane provides the dispersion term in [1]. The stretching modulus near the phase transition is used to determine the nonlinear coefficients a, P, and Q, which are then explained in more detail in the text [1]. Briefly, P represents the position of equilibrium at the phase transition when the membrane is at its softest, Q is the offset in k_a_ (u) space and relates to the calorimetric studies of lipid bilayers (see Fig. 1A in [1]); if c_P_ had no global minimum at the phase transition, Q would be zero. The parameter a is the series expansion coefficient that best fits the observed data in [2]. Its physical meaning is not directly related to the structure of the lipids, but to their thermodynamic properties.

The authors of [1] were unaware of [8] and are therefore grateful to Peets et al. for bringing this publication to our attention. As for the ‘good’ and ‘bad’ Boussinesq-type equation, this was a lapse [1] and the authors of [1] agree with the change of sign.

The discussion of Peets et al. about the unipolarity of solutions can be further clarified by the results of the measurements of Tasaki et al. [9], who only report the change in the height of the membrane as a direct consequence of the volume expansion. This effect can be considered as a non-local phenomenon related to the local change of curvature and not necessarily affecting the soliton propagation. This change is also neglected in the Heimburg–Jackson model [2]. If the difference is that the longitudinal density change in biomembranes can be modeled by a unipolar pulse, but the experimentally measured transverse displacement is not unipolar, this implies that the sign change defining the polarity should be taken into account in our further developments. However, it is not clear how this sign change could be implemented. The fact that transverse and longitudinal displacements are coupled was also not taken into account, since only the transverse displacement was considered in the approximation of the model in [1]. This is appropriate to match the simplicity of the HJ model, which is also one-dimensional, albeit only in the longitudinal direction.

In summary, the comments and discussion of Peets et al. appear to be relevant to improving the technical considerations of future mathematical/physical models related to solitary waves in biological membranes, but not so much to the physical basis of the models presented in [1, 2]. We hope that experimental evidence will soon confirm or refute the predictions of [1]. Nevertheless, the authors of [1] are grateful for the comments of Peets et al. and look forward to new developments in the field of axonal propagation.

## Data Availability

No data associated in the manuscript.
